# A System of Legal Medicine

**Published:** 1894-03

**Authors:** E. B. Treat

**Affiliations:** 5 Cooper Union, New York


A SYSTEM OF LEGAL MEDICINE. A Complete Work of Reference for Medical and Legal Practitioners by Allan McLane Hamilton, M. D., of New York, and Lawrence Godkin, Esq., of the New York Bar, assisted by Thirty Collaborators of recognized ability. In two royal octavo volumes of about 700 pages each. Fully illustrated. In preparation.

The great need of a Standard American Work on Medical Jurisprudence has long been felt; and this work gives abundant promise of being just what the Medical and Legal professions have so long wanted. Every department will be thoroughly and reliably treated.

Further information and advance copy will be forthcoming in due time.

Respectfully yours,

5 Cooper Union, New York. E. B. TREAT.

